# A successful surgical treatment of a closed rupture of flexor digitorum superficialis in surgeon’s hand. A case report and review of literature

**DOI:** 10.1016/j.ijscr.2020.01.041

**Published:** 2020-02-06

**Authors:** Yazeed Alsaadi, Turki S. Alhassan, Mohammed F. Alfawzan, Salah Aldekhayel, Obaid M. Almeshal

**Affiliations:** Department of Plastic Surgery, King Abdulaziz Medical City-Riyadh, Riyadh 11426, P.O. Box 22490, Saudi Arabia

**Keywords:** Tendon injury, Flexor tendon, Tendon avulsion, Tendon rupture, Flexion contracture, Case report

## Abstract

**Introduction:**

Isolated closed rupture or avulsion of the flexor digitomm superficialis (FDS) tendon at its insertion is a rare diagnosis. It can be related to a pathology such as rheumatoid arthritis, bony abnormalities, tenosynovitis, fractures, or tuberculosis.

A review of the literature identified only few cases of closed avulsion or rupture of FDS tendons nonpathologically.

We hope this report will help to gather more experience for the surgical intervention in a delayed presentation of ruptured flexor digitorm superficialis tendon. The work has been reported in line with the SCARE criteria.

**Presentation of case:**

We report a case of 48-year-old surgeon who sustained a trauma to her left middle finger. The patient presented three months after injury with complaints of pain and decreased range of motion of involved digit. Patient was treated conservatively and after failure of conservative treatment surgical intervention was done with complete tendon excision and capsulotomy of Proximal interphalangeal joint.

Patient retained full range of motion and pain subsided.

**Discussion:**

Isolated closed avulsions or rupture of the FDS tendon is a challenging entity in hand surgery in diagnosis and treatment. Nonsurgical treatment with splinting and physiotherapy might help to prevent flexion deformity.

The surgical treatment include tenolysis, flexor digitorum superficialis tendon excision, and in selected patients capsulotomies of involved joints.

**Conclusion:**

A review of the literature identified only few cases of closed avulsion of FDS tendons nonpathologically. Early diagnosis and intervention can prevent sequel of flexion contracture.

## Highlights

• Isolated closed rupture or avulsion of flexor digitorum superficialis tendon is a rare pathology.
• Early diagnosis and managment can prevent irreversible disabilities.
• In our case we demonstrate the clinical presentation and surgical management of a closed ruptured flexor digitorum superficialis.

## 1 Introduction

The aetiology of infrequent rupture of flexor digitorum superficialis compared to profundus is unknown, but smaller insertion area of flexor digitorum profundus can play a role. Also flexor digitorum profundus involvement in hand functions is more that makes it more vulnerable to injury [2].

A review of the literature identified only few cases of closed avulsion of FDS tendons nonpathologically. Boyes et al. reported only three cases [1]. Folmar et al. reported two similar cases [2]. Both did not advocate surgical treatment [1,2].

Thomas et al. reported a case report of a surgically managed isolated flexor digitorum superficialis tendon rupture [3].

Stern et al. reported surgically treated eleven cases [5]. James et al. reported that, the most common affected digit was the ring finger [5].

Mark et al. reported one case and treated surgically [6]. Ferraro et al. reported surgically treated avulsion of the bony insertion of flexor digitorum superficialis tendon [7]. Vandeputte et al. reported a surgically treated closed avulsion of flexor digitorum superficialis with avulsion of anular pulleys [7].

The presentation usually is delayed with a flexion deformity of proximal interphalangeal joint [1,2].

As patient sustain a minor trauma, usually they will not seek a specialized medical advice unless further contracture will lead to a flexion deformity of the digit [5].

Nonsurgical treatment with splinting and physiotherapy might help to prevent flexion deformity [5].

The surgical treatment include tenolysis, flexor digitorum superficialis tendon excision, and in selected patients capsulotomies were done [5].

## 2 Patient information

A 48 years old right handed obstetrician who sustained a minor trauma to her left middle finger.

Patient was seen by general practitioner and avulsion was not diagnosed and discharged on pain medications and according to the chart ROM and tendons conditions were not documented.

Patient visited hand surgery clinic after 3 months from the injury with complaints of pain and decreased range of motion of the involved digit. Patient denied any history of previous hand trauma other than this minor trauma and systematic review was unremarkable with no significant past medical or surgical history.

## 3 Clinical findings

Examination revealed tenderness over the flexor sheath of the involved finger with extension lag of 30 degrees and bulge at the palm over the distal palmar crease.

Passive range of motion at PIPJ and MCPJ was decreased with flexion deformity of the digit.

Normal vascularity and intact sensation of the digit.

## 4 Diagnostic assessment

Plain radiograph was unremarkable with normal bone alignment.

**Fig. 1:**
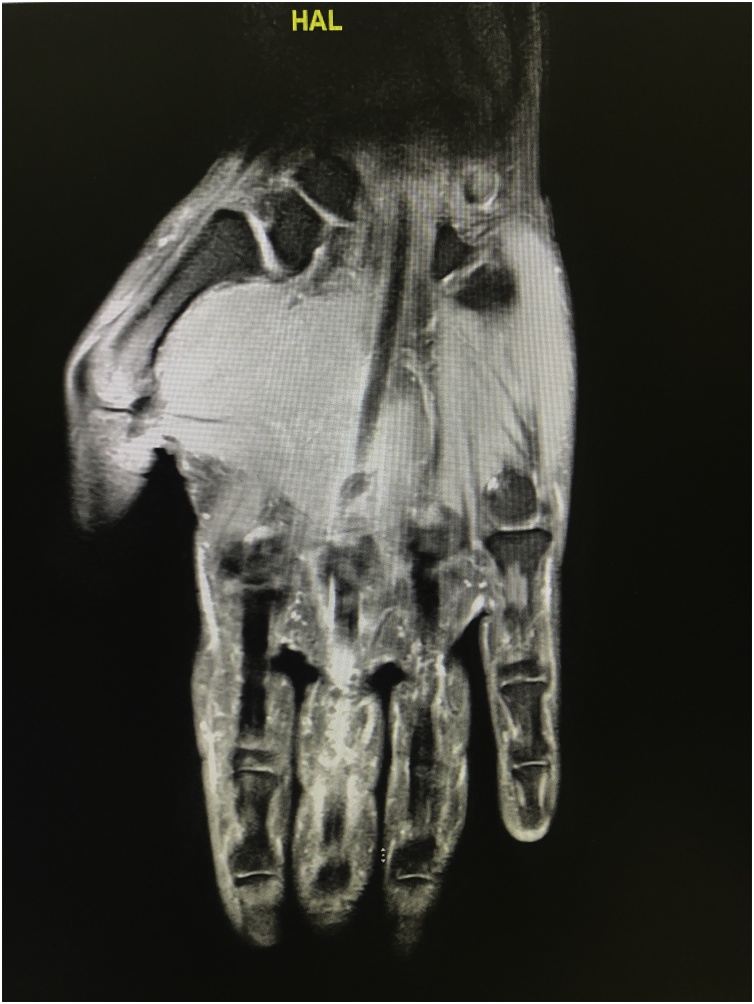
MRI hand showing discontinuty of FDS tendon.

**Fig. 2:**
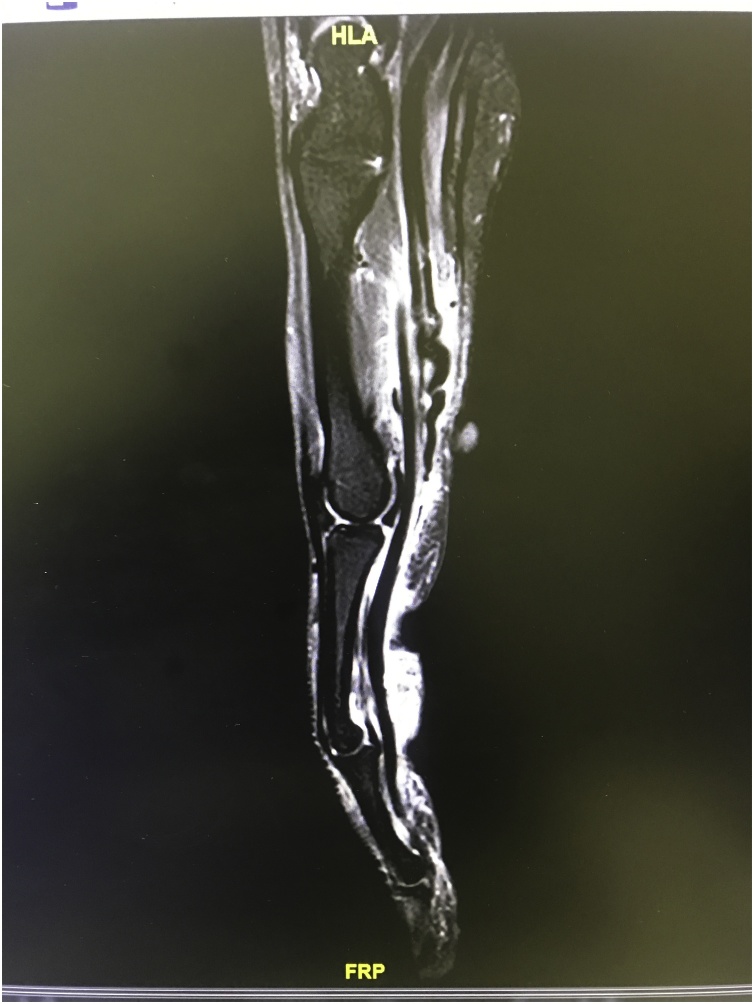
MRI of involved finger showing coiled ruptured FDA tendon.

MRI confirmed ruptured flexor digitorum superficialis with 4.5 cm retraction [Fig. 1, Fig. 2].

## 5 Therapeutic intervention

At time of presentation, patient was referred to physiotherapy for splinting and range of motion exercises. The goal was to improve the deformity and to improve ROM at PIPJ.

After two month of conservative treatment deformity didn’t improve as well as ROM at PIPJ, so surgical treatment was decided.

Surgical Technique:

Under General Anesthesia with Tourniquet control. After preparation under sterile technique procedure done as the following steps:

Step 1: Exploration of the middle finger through a bruner incision was done.

Step 2: Flexor digitorum superficialis tendon was found completely ruptured and retracted with entrapement of flexor digitorum profundus and fibrosis. Compete excision of the ruptured flexor digitorum supergicialis and tenolysis of flexor digitorum profundus was done and passive range of motion was improved but still not full.

Step 3: Open calpsulotomy and release of accessory collateral ligaments were done and full range of motion achieved.

Step 4: Skin closure was done with a z-plasty.

Patient was referred to physiotherapy for range of motion exercises and splinting to keep PIPJ and MCPJ in full extension.

**Fig. 3:**
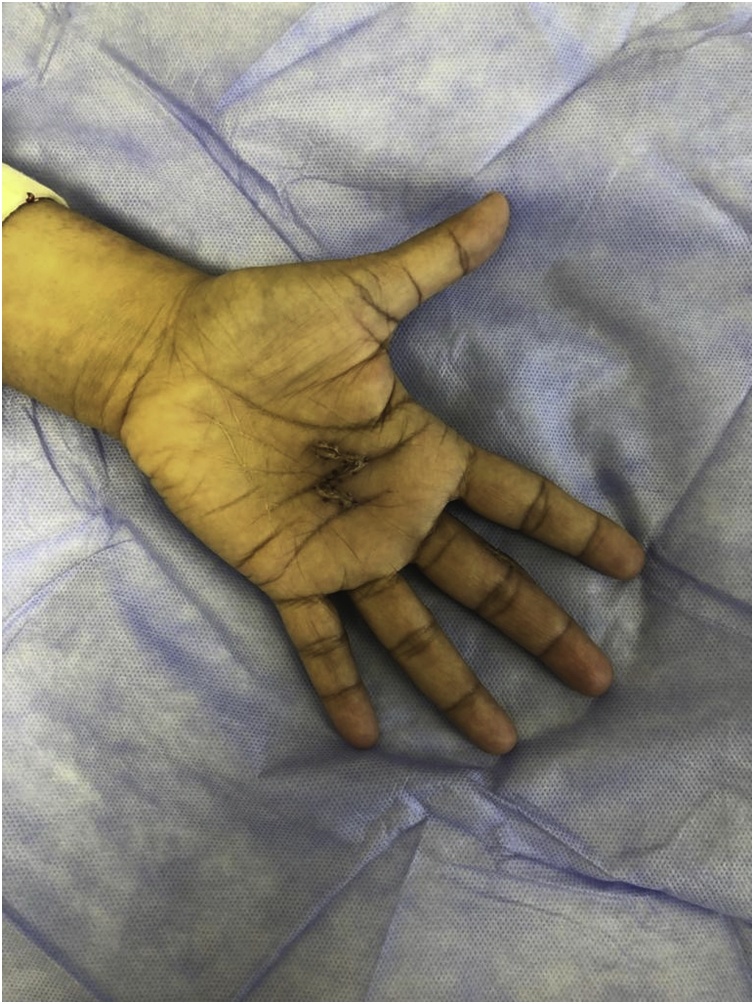
Three weeks postoperatively with full extension at MCPJ and IPJ.

**Fig. 4:**
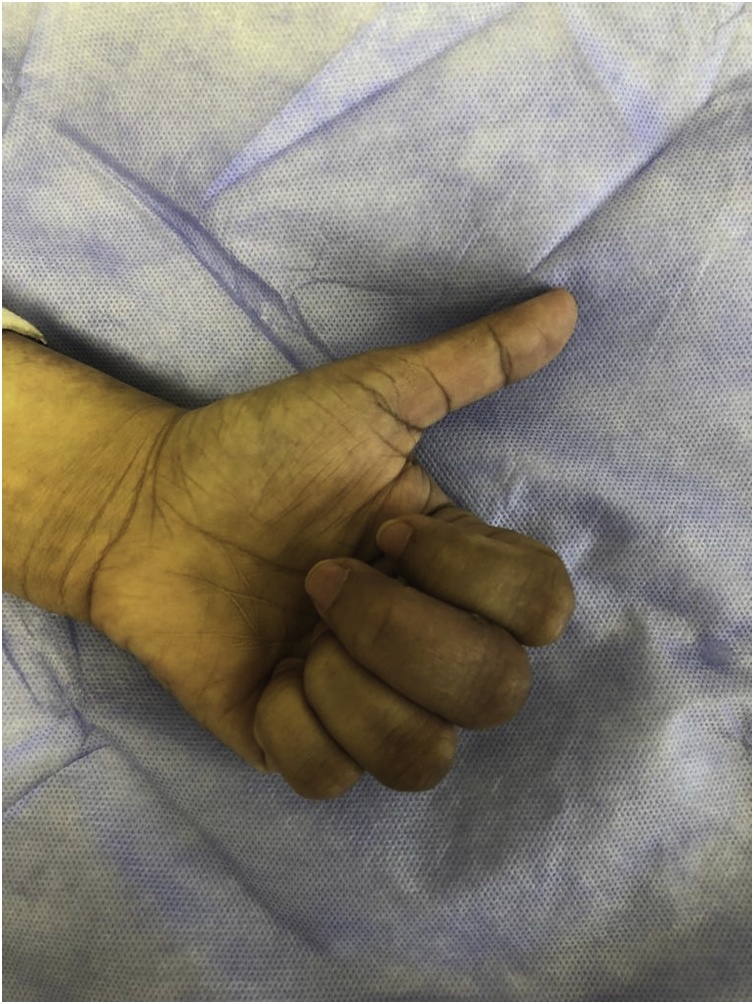
Three weeks post operatively with improvement of MCPJ and IPJ flexion but not Full flexion.

Three weeks after surgery deformity and range of motion was improved [Fig. 3, Fig. 4].

**Fig. 5:**
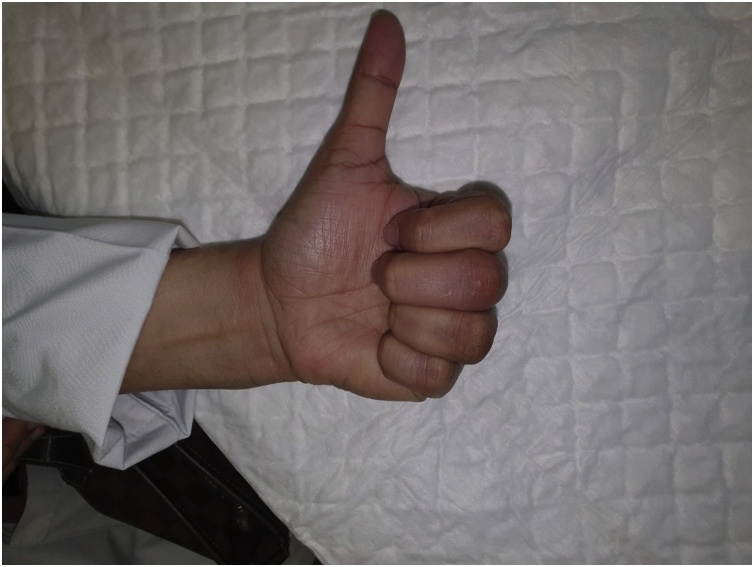
Six months postoperatively showing full flexion at MCPJ and IPJs.

**Fig. 6:**
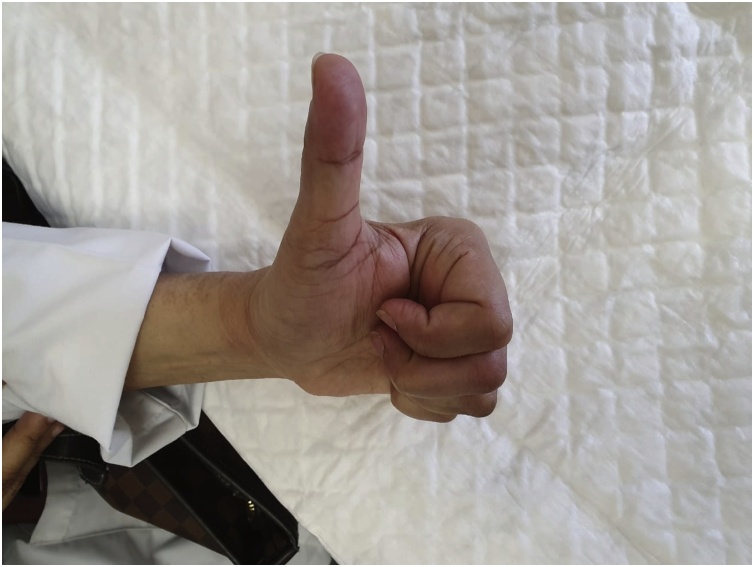
Six months postoperatively showing full flexion at MCPJ and IPJs.

After completion of six months from surgery full ROM without any residual deformity was achieved [Fig. 5, Fig. 6], [video 1 (in Supplementary material)].

## 6 Discussion

Isolated closed avulsions of the FDS tendon at its insertion is a challenging entity in hand surgery in diagnosis and treatment [4]. In our case, the patient was a surgeon and management of the rupture prevented the sequel of flexion contracture. Once tendon avulsed from its insertion at middle phalynx, the FDS tendon retracts proximally. At the champer chiasm FDS will act as noose on FDP which will ensnare it. Entrapement of FDP if not diagnosed and treated early will ultimately lead to adhesions, inflammation and flexion contracture of the PIP joint [4].

Carefull hand examination even after minor trauma is critical not to miss this diagnosis. Conservative treatment in the form of passive and active extension exercises and splinting can prevent subsequent deformity [4]. Surgical intervention should be planned if conservative treatment failed to improve the deformity [5].

Based on the cases reported in the literature, different injury can be seen with avulsion or rupture of flexore digitorum superficialis.

These injuries can be classified anatomically into:

1. Closed avulsion of one slip or both slip of FDS.
2. Closed rupture with intact stump.
3. Closed avulsion of both slip of FDS with annular pulley.
4. Closed avulsion of the bony insertion of FDS.
5. Closed avulsion of both FDS and FDP.

## 7 Conclusion

In summary, Isolated closed avulsion of the flexor digitomm superficialis (FDS) tendon at its insertion may lead to permanent disability. Early diagnosis can prevent finger deformity.

Surgical intervention can achieve good result in patient presenting with flexion deformity.

Physiotherapy before and after surgery found to be important element in the management.

Surgical intervention was needed in most of cases in the literature.

Further studies are needed for the best management of each type of these injuries.

## Declaration of Competing Interest

The authors report no conflicts of interest. The authors alone are responsible for the content and writing of the paper.

## Sources of funding

No fund.

## Ethical approval

This study was approved by the Ethics Committee at King Abdullah medical city. The patient was informed and consented for the publication of this work.

## Consent

Consent obtained from the patient for publication and accompanying pictures.

## Author contribution

Conceptualization: Dr. Obaid Almishal.

Investigation: Dr. Turki Alhassan.

Resources: Dr. Salah Aldekhayel.

Dr. Yazeed Alsaadi: Writing – Original Draft.

Dr. Mohammed Alfawzan: Writing – Review & Editing.

All Authors: Visualization.

Dr. Obaid Almishal: Supervision.

Dr. Yazeed Alsaadi.

Dr. Yazeed Alsaadi: Writing paper, literature review and data collection.

Dr. Turki Alhassan: data collection.

Dr. Mohammed Alfawzan: Editing paper.

Dr. Salah Aldekhayel: Paper reviewer.

Dr. Obaid Almishal: Main Surgeon, clinic follow up and data collection.

## Registration of research studies

Not needed.

## Guarantor

Dr. Yazeed Alsaadi.

## Provenance and peer review

Not commissioned, externally peer-reviewed.

## References

[1] Boyes J.H., Wilson J.N., Smith J.W. (1960). Flexor-tendon ruptures in the forearm and hand. J. Bone Joint Surg. Am..

[2] Folmar R.C., Nelson C.L., Phalen G.S. (1972). Ruptures of the flexor tendons in hands of non-rheumatoid patients. J. Bone Joint Surg. Am..

[3] Agha R.A., Borrelli M.R., Farwana R., Koshy K., Fowler A., Orgill D.P., For the SCARE Group (2018). The SCARE 2018 Statement: Updating Consensus Surgical CAse REport (SCARE) Guidelines. Int. J. Surg..

[4] Cheriyan T., Neuhaus V., Mudgal C. (2012). Isolated closed rupture of the flexor digitorum superficialis tendon. Indian J. Plast. Surg..

[5] Stern J.D., Mitra A., Spears J. (1995). Isolated avulsion of the flexor digitorum superficialis tendon. J. Hand Surg..

[6] Courtney M.J., MacIndo N.L. (2020). Isolated traumatic avulsion of the flexor digitorum superficialis tendon — a case report and review of this condition. Hand Surg..

[7] Ferraro S.P., Schenck R.R. (1998). Isolated closed rupture of the bony insertion of the flexor digitorum superficialis tendon: an unusual case. J. Hand Surg..

